# Liquid‐Based Multijunction Molecular Solar Thermal Energy Collection Device

**DOI:** 10.1002/advs.202103060

**Published:** 2021-09-28

**Authors:** Zhihang Wang, Henry Moïse, Martina Cacciarini, Mogens Brøndsted Nielsen, Masa‐aki Morikawa, Nobuo Kimizuka, Kasper Moth‐Poulsen

**Affiliations:** ^1^ Department of Chemistry and Chemical Engineering Chalmers University of Technology Kemigården 10 Gothenburg 41296 Sweden; ^2^ Department Chemical Engineering University of California Santa Barbara USA; ^3^ Department of Chemistry “U. Schiff,” University of Florence via della Lastruccia 3–13 Sesto Fiorentino (FI) 50019 Italy; ^4^ Department of Chemistry University of Copenhagen Universitetsparken 5 Copenhagen Ø 2100 Denmark; ^5^ Department of Chemistry and Biochemistry Graduate School of Engineering Kyushu University 744 Moto‐oka Nishi‐ku Fukuoka 819‐0395 Japan; ^6^ Center for Molecular Systems (CMS) Kyushu University 744 Moto‐oka Nishi‐ku Fukuoka 819‐0395 Japan

**Keywords:** molecular solar thermal energy storage efficiency, multijunction solar collector, organic photoswitches, solar energy storage

## Abstract

Photoswitchable molecules‐based solar thermal energy storage system (**MOST**) can potentially be a route to store solar energy for future use. Herein, the use of a multijunction **MOST** device that combines various photoswitches with different onsets of absorption to push the efficiency limit on solar energy collection and storage is explored. With a parametric model calculation, it is shown that the efficiency limit of **MOST** concept can be improved from 13.0% to 18.2% with a double‐junction system and to 20.5% with a triple‐junction system containing ideal, red‐shifted **MOST** candidates. As a proof‐of‐concept, the use of a three‐layered **MOST** device is experimentally demonstrated. The device uses different photoswitches including a norbornadiene derivative, a dihydroazulene derivative, and an azobenzene derivative in liquid state with different **MOST**properties, to increase the energy capture and storage behavior. This conceptional device introduces a new way of thinking and designing optimal molecular candidates for **MOST**, as much improvement can be made by tailoring molecules to efficiently store solar energy at specific wavelengths.

## Graphical abstract

Molecular photoswitches can store solar energy by photochemical conversions. This article investigates theoretically what happens to the solar energy capture efficiency if multiple molecular systems optimized for different parts of the solar spectrum are combined. Further, an experimental device that combines three different molecular photoswitches illustrates the concept.

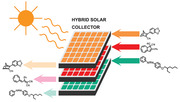

## 1 Introduction

**Figure 1:**
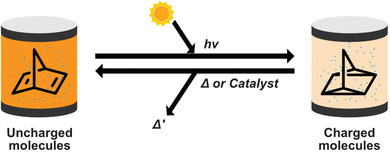
A scheme of **MOST** concept. The photoswitchable molecules (for instance norbornadiene couple) can be switched back and forth through light source and heat/catalyst.

During a year, the sun delivers about 23000 TWy of energy, meaning that it can provide the annual human power need in only seven hours.^[^ ^1^ ^]^ With an increasing population and energy consumption, harvesting a small portion of solar light can potentially relieve the pressure of the worldwide growing energy demand. However, the utilization of solar energy is strongly dependent on both time‐variant and geographical conditions.^[^ ^2^ ^]^ Molecular solar thermal energy storage (**MOST**) is one promising technology that can potentially be used for solar capture and storage purposes.^[^ ^3^ ^]^ The system is made by organic photoswitchable molecules that can capture, store and release solar energy on‐demand.^[^ ^4^ ^]^ The uncharged low energy parent molecule can absorb and store sunlight in the form of chemical energy, thus photoisomerizing to a higher energy, metastable photoisomer. The converted molecule can then be stored ideally over an appropriate period of time. Later, when energy is needed, the charged molecule can be back‐converted by heat or by an efficient catalyst on demand and release the stored chemical energy as heat. (see **Figure** **1**)

**Figure 2:**
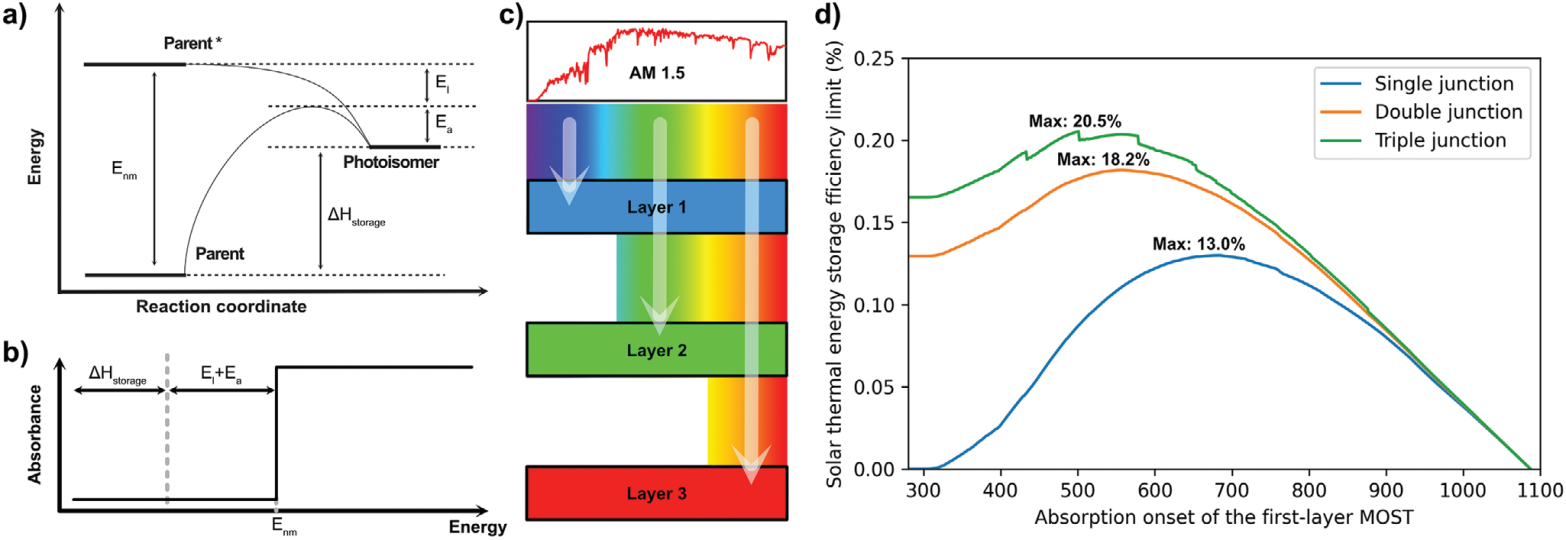
a) Energy diagram of the photoisomerization reaction. *E*_nm_ represents the *S*_0_–*S*_1_ gap of the photoswitch. *E*_l_ is the thermal relaxation loss after photoisomerization reaction. *E*_a_ is the thermal back‐conversion barrier and Δ*H*_storage_ is the energy storage enthalpy of the molecule. b) Absorption profile of a MOST candidate is assumed as a step function. All the incoming photons above the threshold energy (onset of absorption) are fully absorbed. c) Scheme of a triple‐junction MOST collection. d) Estimation of the energy storage efficiency limit of *E*_l_ +   *E*_a_ =  110 kJ mol^−1^ compounds (*τ*_1/2_ = 24 d at 25 °C), in single‐junction system (efficiency limit: 13.0%), in double‐junction system (efficiency limit: 18.2%), and in triple‐junction system (efficiency limit: 20.5%).

From previous research, various key parameters for an optimal **MOST** system have been assessed.^[^ ^5^ ^]^ The whole process can be illustrated by an energy diagram, as shown in **Figure** **2**a. The parent molecule can initially be photoexcited by incoming solar energy (*E*_nm_) to an excited state (parent*), then the molecule will be photoisomerized to the corresponding metastable higher energy photoisomer. In an ideal case here, the photoisomerization quantum yield is assumed as unity to simplify the further calculation. Normally, the photochemical process could generate some relaxation energy loss, noted as *E*_l_, and the energy difference between the photoisomer and its initial parent state is the stored energy in the system (Δ*H*_storage_). In order to release the stored energy, the metastable high‐energy photoisomer needs to overcome an energy barrier (*E*_a_) to initiate the back‐conversion reaction. This energy barrier is thermodynamically related with the energy storage half‐life.

To estimate the efficiency limit of a **MOST** system, with above mentioned parameters, Equation (1) can be formulated^[^ ^5a^ ^]^

(1) $$\begin{array}{c} \eta_{\mathrm{MOST}\_\mathrm{limit}}=\frac{\dot{N}\left(E_{\mathrm{nm}}\right)\cdot \left(E_{\mathrm{nm}}-E_{a}-E_{l}\right)}{{\dot{E}}_{\mathrm{sun}}}=\frac{\dot{N}\left(E_{\mathrm{nm}}\right)\cdot \Delta H_{\mathrm{storage}}}{{\dot{E}}_{\mathrm{sun}}} \end{array}$$

where $\dot{N}$ represent the number of absorbed photons at an excitation energy *E*_nm_ per unit time, and per irradiated area. ${\dot{E}}_{\mathrm{sun}}$ corresponds to the total incoming solar power with air mass 1.5. In this model, the molecular absorption profile is modeled as a step function as shown in Figure 2b. It is assumed that all incoming photons from the solar spectrum above the threshold energy are completely absorbed by the photoswitchable molecules.

For an ideal **MOST** system, it was previously estimated that the maximum solar energy storage efficiency can reach up to 12.4% at an *S*_0_‐*S*_1_ gap of 1.81 eV and a storage time of 24 d.^[^ ^5a^ ^]^ To further enhance this efficiency limit, a multijunction device design, inspired by multijunction solar cells, is an interesting concept to introduce. For single‐junction polymer solar cells, the maximum efficiency was estimated to be around 10–13% with a bandgap of 1.5 eV.^[^ ^6^ ^]^ However, this efficiency can be further increased by changing the single‐junction to multijunction. Based on the quadruple‐junction design, the efficiency has been predicted to reach up to 41.7%.^[^ ^7^ ^]^ Inspired by these device design strategies, similar ideas have been realized by multijunctional **MOST** in solid form with azobenzene molecules.^[^ ^8^ ^]^ For instance, the overall efficiency was measured as 0.4% for a multilayer azobenzene derivative multijunctional film, higher than any azobenzene film‐based **MOST** device to date.

Compared to a solid‐state system, liquid‐based **MOST** systems have the advantage of easy transportation and functioning, e.g., the liquid solution can be pumped in a closed system for automated operating cycles.^[^ ^5^, ^9^ ^]^ Thus, it is stimulating to consider to estimate the efficiency limit change and design of a multilayered flow device. Intuitively, each layer could be flowed with different molecular solutions, which cover different parts of the solar irradiation spectrum (see Figure 2c). Hence, we first start with a parametric study of the limit efficiency difference on liquid‐based multijunction **MOST** setup, then experimentally demonstrate the concept with three different molecular candidates (a norbornadiene derivative **NBD**, a dihydroazulene derivative **DHA**, and an azobenzene derivative **AZO** in liquid state) in a triple‐junction microfluidic device.

## 2 Estimating the Solar Energy Storage Efficiency Limit on Liquid‐Based Multijunction MOST Device

To simplify the calculation, it is assumed that, with solar irradiation started from 300 nm as a cutoff wavelength, the energy loss during photoisomerization reaction *E*_l_ is equal to zero. We also note that this height of *E*_a_ has a large impact on the theoretical limit of the **MOST** system, and is one of the main reasons that the values of $\eta_{\mathrm{MOST}\_\mathrm{limit}}$ in different works are not the same.^[^ ^5a,c^ ^]^ In our study, we consider only an *E*_a_ of 110 kJ mol^−1^ (24 d half‐life at room temperature) to keep the uniformity and simplicity of the calculation.

In order to estimate the effect of using a double‐layer device, we first note the calculated efficiency at each onset wavelength of the first layer as *η*_MOST1_(*E*_nm_), then we introduce to the model a second layer with **MOST** molecule. In this case, all photons that have wavelengths shorter than the onset wavelength of the first layer molecules are completely absorbed by the molecules in the first layer. So the incoming photon's wavelength to the second layer will start from the onset to the end of the solar irradiation spectrum (see Figure 2c). Under such circumstances, we calculate the efficiency limit of the second layer with *n* nm redshifted to the first layer (*n* = 1, 2, 3…until the onset of the second layer corresponds to the end of the solar spectrum), and calculate with each red‐shifted wavelength, its corresponding efficiency limit (note *η*_MOST2_). The maximum efficiency limit of the red‐shifted second layer is then noted as $\eta_{\mathrm{MOST}2_{\max}}\left(E_{\mathrm{nm}}\right)$. Hence, the new overall efficiency limit for this double‐layer system can then be expressed as Equation (2)

(2) $$\eta_{\mathrm{MOST}\_\mathrm{double}-\mathrm{junction}}\left(E_{\mathrm{nm}}\right)\;=\eta_{\mathrm{MOST}1}\;\left(E_{\mathrm{nm}}\right)+\eta_{\mathrm{MOST}2_{\max}}\left(E_{\mathrm{nm}}\right)$$

With Equation (2), the efficiency limit of a double‐layer system can be estimated and plotted as shown in Figure 2d. Clearly, this limit can then be further pushed from 13.0% to 18.2%, with an optimal onset of absorption of 556 nm for the first layer and 760 nm for the second layer.

Similar calculations can be performed for a triple‐junction device estimation. Compared to the double‐layer concept, an additional third layer has been introduced to the model. In such a case, the total efficiency limit can be calculated with Equation (3)

(3) $$\begin{array}{ccc} \eta_{\mathrm{MOST}\_\mathrm{triple}-\mathrm{junction}}\left(E_{\mathrm{nm}}\right) & = & \eta_{\mathrm{MOST}1}\;\left(E_{\mathrm{nm}}\right)+\eta_{\mathrm{MOST}2_{\max}}\left(E_{\mathrm{nm}}\right)\\ & & +\;\eta_{\mathrm{MOST}3_{\max}}\left(E_{\mathrm{nm}}\right) \end{array}$$

A maximum efficiency limit of 20.5% can be hence obtained; the optimal onset of absorption for the first layer is located at 501 nm, 718 nm for the second layer, and 883 nm for the third layer.

To be mentioned, it is also possible to add an infinite number of layers for the calculation. However, the efficiency limit does not significantly change using quadruple or quintuple‐junction design, which converges around 21.2%, indicating that a multijunction **MOST** system can only store a maximum of 21% of the solar power as chemical energy (see S1, Supporting Information for python coding script).

## 3 Proof‐of‐Concept Demonstration of the Liquid‐Based Triple‐Junction MOST Device

**Figure 3:**
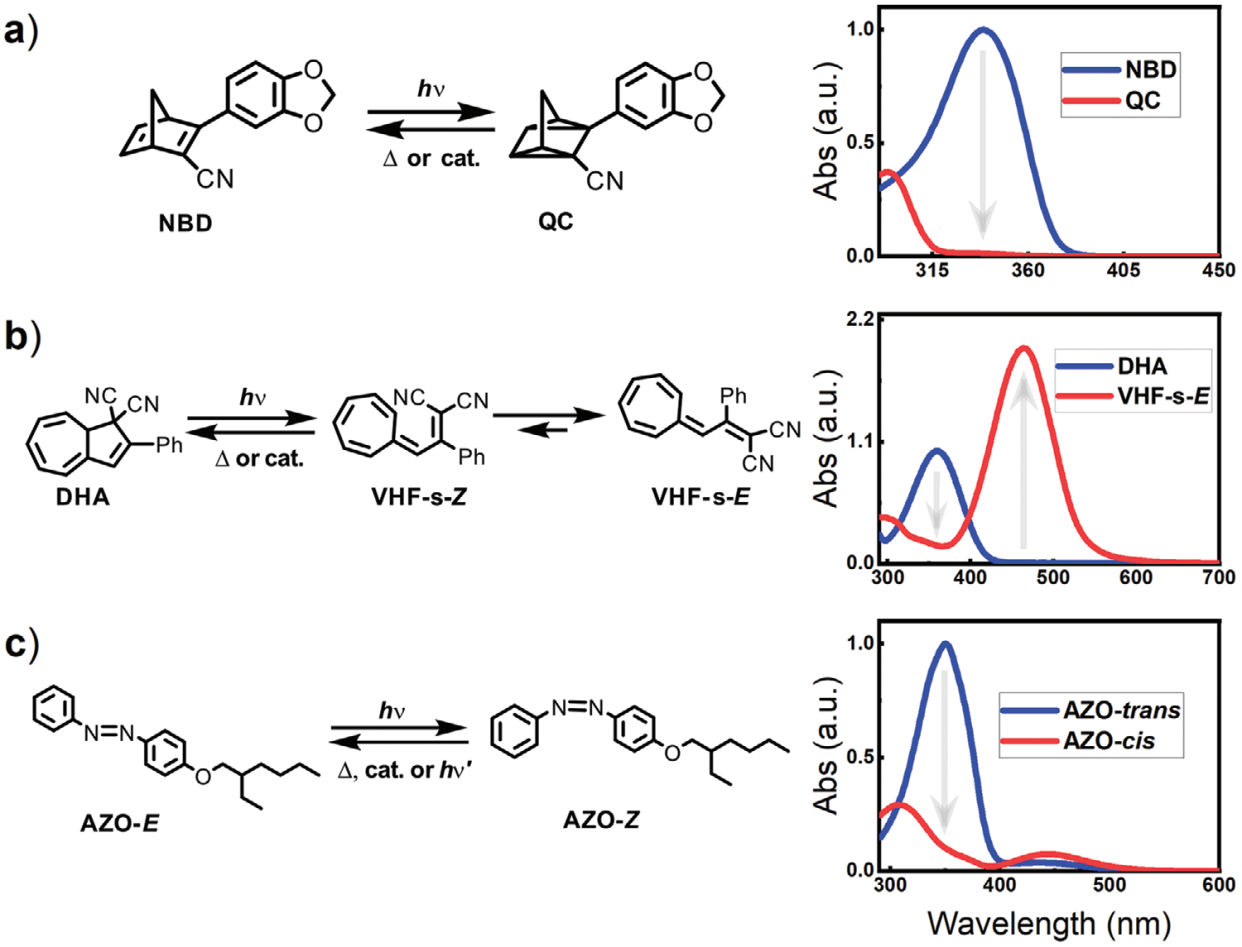
Molecular structure and absorption spectrum of a) **NBD**/**QC** in toluene; b) **DHA**/**VHF** in toluene, and c) **AZO**‐*E*/Z in toluene.

To experimentally illustrate the concept, a liquid‐based triple‐junction **MOST** device has been demonstrated in this work. As the most well‐studied molecular systems, norbornadiene derivatives,^[^ ^10^ ^]^ dihydroazulene derivatives,^[^ ^11^ ^]^ and azobenzene derivatives^[^ ^12^ ^]^ have gathered increasing attention for **MOST** purposes in recent years. As an experimental proof‐of‐concept, three compounds have been selected for demonstration in this work. The corresponding molecular structures in this work are shown in **Figure** **3**. Once exposed under a light source, **NBD** can photoisomerize to its corresponding photoisomer quadricyclane (**QC**) via a [2+2] cycloaddition reaction. The high‐energy, metastable state vinylheptafulvene (**VHF**) is formed via a light‐induced ring‐opening of its parent state **DHA**. **VHF** exists in an equilibrium between s‐*Z* and s‐*E* conformers, the latter being the most stable. **AZO** can be photoconverted from the initial *E*‐state to its high‐energy *Z* form. With different switching mechanisms, these three candidates feature different photochemical properties in toluene (summarized in **Table** **1**). It can be seen that the onset of **NBD** absorption is located at 383 nm. With a photoisomerization quantum yield of 82%, its photoisomer **QC** has almost zero absorption overlap with the parent state after conversion. Compared to **NBD**, the advantage of **DHA** and **AZO** consists of further redshifted absorption spectra with onset at 452 and 514 nm, respectively.

**Table 1:** Photophysical data of used photoswitchable candidates in this study

	*λ*_onset_ [nm]	*λ*_max_ [nm]	*ε*_max_ [M^−1^ cm^−1^]	Φ_iso_ [%]	*t*_1/2_ [h]	Δ*H*_storage_ [kJ mol^−1^]	*η*_max_ [%]
**NBD**	383	338	1.2 × 10^4^	82	47	95	0.79
**DHA**	454	360	1.6 × 10^4^	60	25	35	0.81
**AZO**	514/400^a)^	350	2.5 × 10^4^	21	36	52	0.88/0.12^a)^

^a)^ Onset of absorption is defined as the wavelength where the absorptivity of the molecule is close to 100 M^−1^ cm^−1^. For **AZO** *‐E*, the onset of absorption is located at 514 nm by definition; however, **AZO‐** *Z* can also be back‐converted to *E* form at such wavelength to finally reach a photostationary state. Its actual *π*–π* conversion onset wavelength can also be defined around 400 nm. In such case, *η*_max_ will be drastically decreased by calculation which equals 0.12%.

Theoretically, to estimate the maximum solar energy conversion efficiency of a specific molecule, the following Equation (4) can be used^[9,12b]^

(4) $$\eta_{\mathrm{MOST}}=\frac{\int_{0}^{\lambda_{\mathrm{onset}}}\frac{E_{\mathrm{AM}\;1.5}\left(\lambda \right)\cdot \left(1-T\right)\cdot \phi_{\mathrm{iso}}\cdot \Delta H_{\mathrm{storage}}}{h\nu \cdot N_{A}}\times d\lambda }{\int E_{\mathrm{AM}\;1.5}\left(\lambda \right)d\lambda }\;\times 100\%$$

where *E*_AM 1.5_(*λ*) represents the solar spectral irradiance in J s^−1^ m^−2^ nm^−1^, *T* corresponds to the transmittance of the used molecule in a specified device, *φ*_iso_ is the photoisomerization quantum yield, Δ*H*_storage_ represents the energy storage enthalpy in kJ mol^−1^, *h* is the Plank constant in J s, *ν* denotes the frequency of incoming light in s^−1^, and *N*_A_ is Avogadro's constant. Hence, it can be calculated that, for each candidate with its corresponding onset of absorption conversion photoisomerization quantum yield and Δ*H*_storage_ shown in **Table** **1**, the maximum energy storage efficiency is 0.79% for **NBD**, 0.81% for **DHA**, and 0.88% for **AZO**.

Experimentally, the optimum energy storage efficiency is defined as, the efficiency under a particular residence time of the fluid in a device, where a maximum conversion percentage is reached.^[^ ^5d^ ^]^ To experimentally determine this value, three 1 × 10^−3^ m solutions of **NBD**, **DHA**, and **AZO** were prepared in toluene. Each solution was passed through a single‐layer microfluidic chip with different flow speeds.

Because of the spectrum overlap between **VHF**‐s‐*E* and **DHA**, **AZO**‐*Z* and *E* states, the parent molecular concentration under different residence times in the chip cannot be directly calculated via Lambert's law. Hence, the following Equation (5) needs to be used^[^ ^12b^ ^]^

(5) $$C_{\mathrm{parent}}=\frac{\frac{A_{\lambda }}{l}-\frac{\varepsilon_{\mathrm{photoisomer}}\left(\lambda \right)\cdot A_{\mathrm{isosbestic}}}{\varepsilon_{\mathrm{isosbestic}}\cdot l}}{\varepsilon_{\mathrm{parent}}\left(\lambda \right)-\varepsilon_{\mathrm{photoisomer}}\left(\lambda \right)}\;$$

where *A*_*λ*_ represents the unitless experimental absorbance during conversion experiment; *l* is the optical pathlength of the chip in cm; *ε*_parent_(*λ*) and *ε*_photoisomer_(*λ*) correspond to the absorptivity of the parent molecule and the photoisomer at wavelength *λ* in M^−1^ cm^−1^, respectively; *A*_isosbestic_ is the unitless experimental absorbance of molecular isosbestic point; *ε*_isosbestic_ represents the absorptivity of the isosbestic point in M^−1^ cm^−1^.

**Figure 4:**
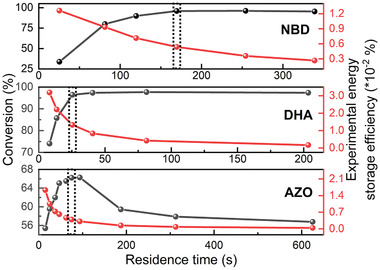
Experimental conversion percentage and energy storage efficiency of **NBD** versus residence time in chip (top figure); **DHA** versus residence time in chip (middle figure); **AZO** versus residence time in chip without bandpass filter (bottom figure). Dashed areas represent the selected optimum energy storage efficiency at maximum conversion percentage, 0.005% with 96% conversion for **NBD**, 0.013% for 96% conversion for **DHA**, and finally 0.004% for 66% conversion of **AZO**. Each data point was recorded three times with multiacquisition counts per second (see S4, Supporting Information for detailed data acquisition and analysis)

To calculate the experimental energy storage efficiency with different solution residence time in chip, Equation (6) can be used^[^ ^11a^ ^]^

(6) $$\eta_{\mathrm{MOST}}=\frac{{\dot{n}}_{\mathrm{parent}}\cdot \alpha_{\mathrm{photoisomer}}\cdot \Delta H_{\mathrm{storage}}}{A\cdot E_{\mathrm{AM}\;1.5}}$$

where ${\dot{n}}_{\mathrm{parent}}$ is the flow speed of parent molecular solution in mol s^−1^, *α*_photoisomer_ corresponds to the conversion percentage, and *A* is the effective irradiated surface in m^2^. Based on this equation, it can be shown that, with different residence times in a single layer device (see S2, Supporting Information for details of microfluidic chips), which also correspond to the fluid exposure time under solar irradiation, the optimum efficiency at near 100% conversion can reach 0.005% with 96% conversion for **NBD**, 0.013% for 96% conversion for **DHA**, and finally 0.009% for 66% conversion of **AZO**. To be mentioned, the measured energy storage efficiency of **AZO** first increased then further decreased with the sample residence time in the device. This is likely due to the unperfect filtration capacity of the used bandpass filter, i.e., when the exposure time increases, more visible photons can penetrate the bandpass filter, thus back‐converting **AZO**‐*Z* molecules (see **Figure** **4**, S3, Supporting Information).

**Figure 5:**
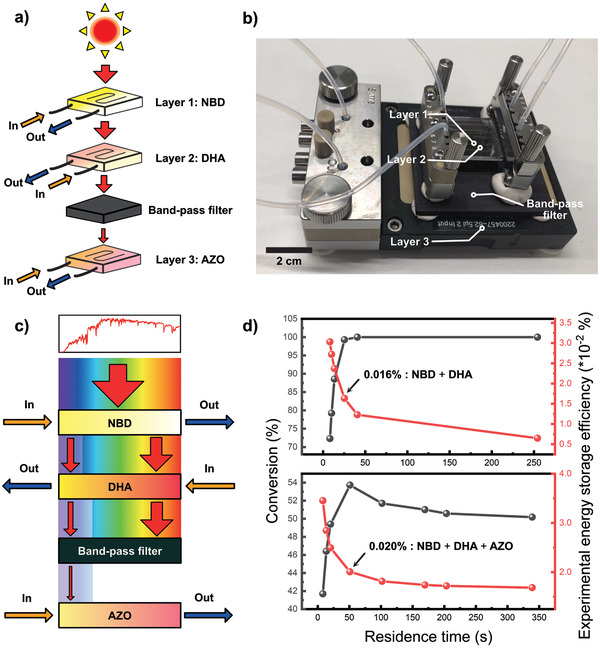
a) The triple‐junction MOST device with three different photoswitchable candidates; **NBD** was set to flow in layer 1, **DHA** was set to flow in layer 2 with the counter flow direction of chip; The band‐pass filter will filter the residual light to convert **AZO** in layer 3. The flow direction in layer 3 is the same as in layer 1. b) Actual photo of the triple‐layer device. c) Scheme of the solar intensity (red arrows) after each layer of the device. d) Experimental conversion percentage and energy storage efficiency of the **NBD** and **DHA** layers (top Figure) and the whole device (bottom Figure). With a maximum conversion percentage, a total energy storage efficiency of 0.020% can be obtained for the hybrid device. Each data point was recorded three times with multiacquisition counts per second (see S4, Supporting Information for detailed data acquisition and analysis).

Unfortunately, the optimum solar energy storage efficiency of a single chip device can only reach around 0.01%. This large difference with the theoretical estimated limit is because of long residence time in device, low concentrated solutions (1 × 10^−3^), and internal photon loss due to the spectral overlap of parent and photoisomer molecules during the conversion process as well as the molecules not capturing all photon energy due to quantum yields that a less than 100% and storage energies lower than the photon energy.^[^ ^11a^ ^]^ In order to capture more residual solar energy, thus increase the solar thermal energy storage efficiency to demonstrate the multijunction concept, we have set up a triple‐junction solar thermal energy storage device with all those three photoswitches. According to the absorption profile of the three couples, it is desirable to set the **NBD** solution as the first layer of the fluidic solution. The second fluidic layer was set up with **DHA** solution. To be able to capture more residual light after the first two layers, **AZO**‐based solution was put on the bottom of the device. It should be noticed from the single‐chip experiments that **NBD** and **DHA** can easily reach near 100% conversion; however, the maximum conversion percentage of **AZO** can only attain 66%. This is mainly due to the photoinduced back‐conversion of **AZO** under visible light. Therefore, to reduce this challenge, a bandpass filter has been inserted between layer two and layer three. (see **Figure** **5**a–c, Figure S3, Supporting Information)

Experimentally, the first layer of **NBD** solution was flowing with a speed of 12 µL min^−1^ to maintain the near‐complete conversion to **QC** and optimum solar storage efficiency of 0.005%. The second layer was then tested with a different flow speed of **DHA** to obtain the optimum flow speed. At this stage, in order to maximize the utilization of incoming photons, the flow direction was set as opposed to the first layer. Hence, an optimum total of two layers solar energy storage efficiency of 0.016% was measured. By keeping this flow speed in layer 2, layer 3 was also tested with various fluidic residence times, keeping the same flow direction as in layer 1. As a result, an overall energy storage efficiency of 0.020% can finally be determined. Such efficiency is higher than the efficiency of any of the single‐layer devices in this work, and successfully demonstrated the concept of using a multijunction device to increase the overall solar thermal energy storage efficiency (see Figure 5d).

## 4 Conclusion

In summary, we have evaluated the possibility of using a multijunction **MOST** to enhance the solar thermal energy storage efficiency. Theoretically, we estimated the efficiency limit of a double‐ and triple‐junction **MOST** system with two and three ideal **MOST** candidates (assuming a back‐conversion barrier *E*_a_ = 110 kJ mol^−1^, half‐life of 24 d), showing that for double‐junction device, the efficiency limit can reach up to 18.2% with ideal onset of absorption at 556 nm from the first layer and 760 nm from the second layer. For triple‐junction device, this efficiency limit can attain up to 20.5% with an ideal onset at 501 nm from the first layer, 718 nm from the second layer, and 883 nm from the third layer. Additional layers will not add much to the efficiency, since the minimal photon energy that the molecules can absorb is defined in our model by the sum of the *E*_a_ and *E*_l_. This puts an upper limit to the wavelengths that can be utilised. Experimentally, we demonstrated, for the first time, a liquid‐based **MOST** triple‐junction solar collector device. By using 1 × 10^−3^ m solution in toluene of three different photoswitchable molecules, an overall optimum solar energy storage efficiency of up to 0.020% has been measured, higher than any of the single‐junction device studied in this work. Thus, the result shows that, besides seeking an ideal **MOST** molecule which could fulfill all **MOST** criteria, it is also possible to enhance the solar energy storage efficiency by combining **MOST** systems optimized for operation at different wavelengths. We note that photoswitches operating in the NIR traditionally have been difficult to achieve but that very recent demonstrations using **AZO**‐based photoswitches shows that this is possible.^[^ ^13^ ^]^

## Conflict of Interest

The authors declare no conflict of interest.

## Supporting information

Supporting Information

## Data Availability

Relevant data are supplied in the article and in the Supporting Information.
