# Supplementary material for: Liquid‐Based Multijunction Molecular Solar Thermal Energy Collection Device
Source: Adv Sci (Weinh). 2021 Sep 28;8(21):2103060. doi: 10.1002/advs.202103060 (PMC8564455; doi:10.1002/advs.202103060)
Supplement: Supplementary file 1 — Supporting Information [file ADVS-8-2103060-s001.pdf]

© 2021 Wiley-VCH GmbH

ADVANCED SCIENCE
Open Access

Supporting Information

for *Adv. Sci.*, DOI: 10.1002/advs.202103060

Liquid Based Multi-Junction Molecular Solar Thermal Energy Collection Device

*Zhihang Wang, Henry Moïse, Martina Cacciarini, Mogens Brøndsted Nielsen, Masa-aki Morikawa, Nobuo Kimizuka and Kasper Moth-Poulsen**

Supporting Information

Liquid Based Multijunction Molecular Solar Thermal Energy Collection Device

*Zhihang Wang, Henry Moïse, Martina Cacciarini, Mogens Brøndsted Nielsen, Masa-aki Morikawa, Nobuo Kimizuka and Kasper Moth-Poulsen**

Table of content:

S1. Python script of the solar energy storage efficiency limit on liquid-based multi-junction

S2. Microfluidic chip

S3. Bandpass filter

S4. Data acquisition and analysis of experimental energy storage efficiency

S1. Python script of the solar energy storage efficiency limit on liquid-based multi-junction

```
import pandas as pd

import numpy as np

import matplotlib.pyplot as plt


Na = 6.022*10**23


photon_flux = pd.read_csv(r"C:\Users\ Desktop\Python\AM15.csv")


########### ########### ########### ########### ###########Air mass 1.5 data is downloaded from : https://www.nrel.gov/grid/solar-resource/spectra-am1.5.html
########### ########### ########### ########### ########### ###########


wavelength = photon_flux.iloc[:, 0]

Nb_of_photon = photon_flux.iloc[:, 1]


table = pd.DataFrame({'wavelength':wavelength, 'Number_of_phton':Nb_of_photon})

table.head()


re1 = [[],[]]

data = [[],[]]



for i in wavelength:

    data[0].append(i)

    re1[0].append(i)

for i in Nb_of_photon:

    data[1].append(i)


k=1
```

```
for i in range(len(data[1])):
    s = np.sum(data[1][0:k])
    Total_photon = s
    result = s/Na*(Na*(6.626*10**-34)*(2.998*10**8)/(10**-9)/data[0][i]-110000)/1000
    re1[1].append(result)
    k = k+1



re0 = [[],[]]


for i in re1[1]:
    if i >= 0:
        re0[0].append(re1[0][re1[1].index(i)])
        re0[1].append(i)


plt.plot(re0[0], re0[1], label='Single junction')
plt.axis([280, 1100, 0, 0.25])


print("########################################")
print("For double layer MOST, the efficient limit is:", "{:.2f}".format(np.max(re0[1])*100),
"%")
print("With first the layer MOST onset of :", re0[0][re0[1].index(np.max(re0[1]))], "nm")
print("########################################")


##############################################
find_the_max_efficiency_and_wavelength
#################################################


def find_the_max_efficiency_and_wavelength (lis):
    table_transform1 = []
    table_transform2 = []
```

```
    for i in lis:
         table_transform1.append(i[0])
         table_transform2.append(i[1])
    index_trans = table_transform2.index(max(table_transform2))
    if max(table_transform2) >= 0:
        return (table_transform1[index_trans],table_transform2[index_trans])
    else:
        return (1600,0.1*10**-9)
 ################################################ second layer efficiency_limit
########################################################


def efficiency_limit (offset, onset):
    table = []
    index1 = data[0].index(offset)
    index2 = data[0].index(onset)
    total_photon = sum(data[1][index1: index2])
    result = total_photon/Na*(Na*(6.626*10**-34)*(2.998*10**8)/(10**-9)/data[0][index2]-
110000)/1000
    table.append(data[0][index2])
    table.append(result)


    return table


############################################### red shift find max
##########################################################################


def red_shifted(offset):
    re2 = []


    if offset < 1700:
        for j in range(1,1700):
```

```
            if offset+j <= 1700:
                efficiency = efficiency_limit(offset, offset+j)
                re2.append(efficiency)
            else:
                break
        return find_the_max_efficiency_and_wavelength(re2)
        re2 = []

################################################################# double layer calculation final ########################################


local_max = []
second_wave = [[],[]]
double_layer = [[],[]]


for i in re0[0]:


    local_max.append(np.max(red_shifted(i)[1]+re0[1][re0[0].index(i)]))
    double_layer[0].append(i)
    double_layer[1].append(np.max(red_shifted(i)[1])+re0[1][re0[0].index(i)])


plt.plot(double_layer[0],double_layer[1], label='Double junction')
plt.axis([280, 1100, 0, 0.25])
first_legend = plt.legend(loc='upper right')


print("########################################")
print("For double layer MOST, the efficient limit is:", "{:.2f}".format(np.max(local_max)*100), "%")
print("With first the layer MOST onset of :", re0[0][double_layer[1].index(np.max(local_max))], "nm")
print("And with the sencond layer MOST onset of :",red_shifted(re0[0][double_layer[1].index(np.max(local_max))])[0], "nm")
```

```
print("########################################")

################################################################# triple layer
########################################



triple_layer = [[],[]]


for i in re0[0]:
    second_wave[0].append(red_shifted(i)[0])
    second_wave[1].append(red_shifted(i)[1])


for i in second_wave[0]:
    triple_layer[0].append(red_shifted(i)[0])
    triple_layer[1].append(red_shifted(i)[1])


final = [[],[]]


for i in range((len(re0[0]))):
    final[0].append(re0[0][i])
    final[1].append(re0[1][i] + second_wave[1][i] + triple_layer[1][i])


plt.plot(final[0],final[1], label='Triple junction')
plt.axis([280, 1100, 0, 0.25])
plt.xlabel("Absorption onset of the first-layer MOST (nm)")
plt.ylabel("Solar thermal energy storage fficiency limit (%)")
first_legend = plt.legend(loc='upper right')
print("########################################")
print("For triple layer MOST, the efficient limit is:", "{:.2f}".format(np.max(final[1])*100),
"%")
print("With first the layer MOST onset of :", re0[0][final[1].index(np.max(final[1]))], "nm")
```

```
print("And with the sencond layer MOST onset of :",red_shifted(re0[0][final[1].index(np.max(final[1]))])[0], "nm")

print("And with the third layer MOST onset of :",red_shifted(red_shifted(re0[0][final[1].index(np.max(final[1]))])[0])[0], "nm")

print("########################################")


########### ########### ########### ########### ########### more layers ########### ########### ########### ########### ########### ###########


four_layer = [[],[]]


for i in triple_layer[0]:

    four_layer[0].append(red_shifted(i)[0])

    four_layer[1].append(red_shifted(i)[1])


five_layer = [[],[]]


for i in four_layer[0]:

    five_layer[0].append(red_shifted(i)[0])

    five_layer[1].append(red_shifted(i)[1])


final0 = [[],[]]


for i in range((len(re0[0]))):

    final0[0].append(re0[0][i])

    final0[1].append(re0[1][i] + second_wave[1][i] + triple_layer[1][i] + four_layer[1][i])


plt.plot(final0[0],final0[1], label='Quadruple junction')

plt.axis([280, 1100, 0, 0.25])

first_legend = plt.legend(loc='upper right')

final1 = [[],[]]
```

```
for i in range((len(re0[0]))):
    final1[0].append(re0[0][i])
    final1[1].append(re0[1][i] + second_wave[1][i] + triple_layer[1][i] + four_layer[1][i] + five_layer[1][i])


plt.plot(final1[0],final1[1], label='Quintuple junction')
plt.axis([280, 1100, 0, 0.25])
first_legend = plt.legend(loc='upper right')
plt.savefig("MOST multijunction efficiency limit.png", dpi=300)
```

S2. Microfluidic chip

The microfluidic chip used for **NBD** and **DHA** conversion experiments was home designed and made from quartz, its microfluidic channels are etched isotopically (wet) 100 μm depth into the substrate, i.e. the actual optical pathlength have a lateral bias of 100 μm. The chip has a total volume of 33.9 mm^3.

The microfluidic chip for **AZO** made from fused silica was purchased from Syrris Ltd with a total volume of 62.5 μL; it contains an effective exposure volume of 13.6 μL, irradiation surface of 1.6 cm^2 and a channel depth of 85 μm. During the experimental demonstration, all fluid speed was controlled by a pump from the same company.

S3. Bandpass filter

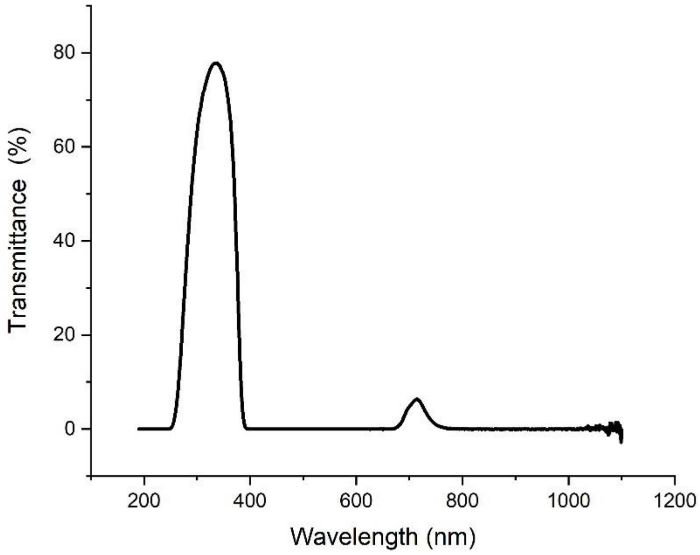

Figure S3. Transmittance spectrum of the bandpass filter used in this study.

S4. Data acquisition and analysis of experimental energy storage efficiency

The experimental conversion setup with the single chip is showing below[1]:

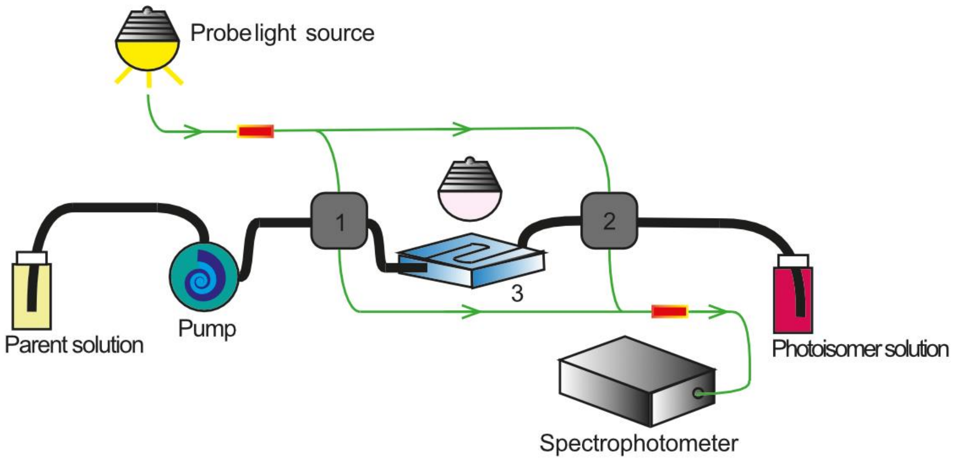

Figure S1. Single-chip experimental setup.

Before running the experiment, the solar simulator above the chip is calibrated to AM1.5 solar irradiation spectrum.

During experiments, the parent molecular solution was pumped with different residence times inside the microfluidic chip. The absorption spectrum of the solution before and after exposure inside the chip can be measured by two separated flow cells 1 and 2 in figure S1, which are connected with a UV-Vis spectrophotometer (AvaSpec-2048L). The conversion percentage can then be calculated by these spectra obtained (each conversion spectrum with different residence time was recorded three times with multi-acquisition counts per second), hence being used later for experimental energy storage efficiency with equation (6) in the main text.

The calculated conversion percentage and calculated energy storage efficiency for single-chip experiments are showing below:

	Residence time (s)	Conversion percentage (%)<br><br>Calculated with UV-Vis spectrometer	Energy storage efficiency (%)<br>Calculated with equation (6)
**NBD**	339	95.30	0.00267

	254	96.17	0.00359
	170	95.95	0.00538
	120	89.87	0.00714
	81	79.91	0.00933
	25	33.75	0.01261
**DHA**	203	97.38	0.00168
	81	97.67	0.0042
	41	97.35	0.00838
	25	96.45	0.01328
	14	85.73	0.02213
	8	74.04	0.03185
**AZO**	625	56.77	4.01E-04
	313	57.89	8.19E-04
	188	59.44	0.0014
	94	66.28	0.00312
	75	66.21	0.0039
	63	65.51	0.00463
	47	65.05	0.00613
	38	61.99	0.00731
	25	59.55	0.01053
	15	55.39	0.01632

For double or triple layer experiments, additional chips were put on the bottom of the first or second layer successively. The calculated conversion percentage and energy storage efficiency for double (**NBD**+ **DHA**) chips and triple chips (**NBD**+ **DHA**+ **AZO**) experiments are showing below:

	Residence time (s)	Conversion percentage (%)<br><br>Calculated with UV-Vis spectrometer	Energy storage efficiency (%)<br>Calculated with equation (6)
**NBD**	170	95.95	0.00538
**NBD + DHA**	254	100.00	0.00648
	41	100.00	0.01228
	25	99.30	0.01634
	14	88.54	0.0237
	10	79.23	0.02724
	8	72.30	0.03031
**NBD**	339	50.19	0.01686

+ DHA + AZO	203	50.58	0.01722
	170	51.01	0.01741
	102	51.70	0.01814
	51	53.72	0.02008
	20	49.40	0.02494
	14	46.41	0.02847
	8	41.69	3.45E-02

[1] Z. Wang, J. Udmark, K. Börjesson, R. Rodrigues, A. Roffey, M. Abrahamsson, M. B. Nielsen, K. Moth-Poulsen, *ChemSusChem* **2017**, *10* (15), 3049
